# Supplementary material for: Effects of Polycyclic Aromatic Hydrocarbons on the Composition of the Soil Bacterial Communities in the Tidal Flat Wetlands of the Yellow River Delta of China
Source: Microorganisms. 2024 Jan 11;12(1):141. doi: 10.3390/microorganisms12010141 (PMC10820892; doi:10.3390/microorganisms12010141)
Supplement: Supplementary file 1 [file microorganisms-12-00141-s001.zip › microorganisms-2750472-supplementary.pdf]

Table S1 The relative abundance (top 10) of soil bacterial communities in in two wetlands in yellow river delta of China.

| | P (%) | CK (%) |
|---|---|---|
| **Phyla** | | |
| Proteobacteria | 36±3.8a | 38±6.5a |
| Actinobacteriota | 11±2.0a | 9±1.6a |
| Chloroflexi | 7±1.8a | 8±3.4a |
| Bacteroidota | 8±3.8b | 15±2.3a |
| Acidobacteriota | 6±2.3a | 1±0.7b |
| Gemmatimonadota | 7±1.9a | 4±0.2b |
| Desulfobacterota | 5±2.2b | 8±0.4a |
| Firmicutes | 3±2.2b | 8±1.8a |
| Myxococcota | 2±0.6a | 1±0.4a |
| unclassified | 5±1.9a | 1±0.3b |
| **Class** | | |
| Gammaproteobacteria | 20±2.8a | 17.1±2.2b |
| Alphaproteobacteria | 16±2.0a | 20.7±6.5a |
| Bacteroidia | 6±2.9a | 4.0±1.9b |
| Anaerolineae | 5±2.1b | 11.4±2.1a |
| Actinobacteria | 6±0.1a | 6.8±2.7a |
| Thermoanaerobaculia | 4±1.2a | 6.0±1.6a |
| Desulfobulbia | 3±1.1a | 0.9±0.5b |
| Desulfuromonadia | 1±1.4a | 0.7±0.4a |
| Rhodothermia | 3±1.2a | 2.4±1.5a |
| Unclassified | 12±2.2a | 2.5±1.7b |
| **Genera** | | |
| *Woeseia* | 4.6±1.0a | 0.1±0.0b |
| *Dadabacteriales* | 1.5±0.4a | 0.4±0.1b |
| *Limibacillus* | 1.1±0.7a | 0.3±0.1b |
| *Halofilum* | 1.7±0.1a | 0.3±0.1b |
| *Rhodovibrio* | 1.1±0.2a | 0.1±0.0b |
| *Sulfurovum* | 1.0±0.1a | 0.1±0.0b |
| *Methylophaga* | 0.9±0.3a | 0.3±0.0b |
| *Desulfobulbusc* | 0.8±0.0a | 0.4±0.0a |
| *Muricauda* | 0.5±0.0a | 0.5±0.1a |
| *unclassified* | 37.6±3.2a | 35.4±4.7a |
